# Independent, external validation of clinical prediction rules for the identification of extended-spectrum β-lactamase-producing Enterobacterales, University Hospital Basel, Switzerland, January 2010 to December 2016

**DOI:** 10.2807/1560-7917.ES.2020.25.26.1900317

**Published:** 2020-07-02

**Authors:** Isabelle Vock, Lisandra Aguilar-Bultet, Adrian Egli, Pranita D Tamma, Sarah Tschudin-Sutter

**Affiliations:** 1 Division of Infectious Diseases & Hospital Epidemiology, University Hospital Basel, University Basel, Basel, Switzerland; 2 Division of Clinical Bacteriology and Mycology, University Hospital Basel, University Basel, Basel, Switzerland; 3 Applied Microbiology Research, Department of Biomedicine, University of Basel, Basel, Switzerland; 4 Department of Pediatrics, Division of Pediatric Infectious Diseases, Johns Hopkins University School of Medicine, Baltimore, United States; 5 Department of Clinical Research, University Hospital Basel, Basel, Switzerland

**Keywords:** Extended-spectrum beta-lactamase-producing Enterobacterales, prediction, validation, ESBL

## Abstract

**Background:**

Algorithms for predicting infection with extended-spectrum β-lactamase-producing Enterobacterales (ESBL-PE) on hospital admission or in patients with bacteraemia have been proposed, aiming to optimise empiric treatment decisions.

**Aim:**

We sought to confirm external validity and transferability of two published prediction models as well as their integral components.

**Methods:**

We performed a retrospective case–control study at University Hospital Basel, Switzerland. Consecutive patients with ESBL-producing *Escherichia coli* or *Klebsiella pneumoniae* isolated from blood samples between 1 January 2010 and 31 December 2016 were included. For each case, three non-ESBL-producing controls matching for date of detection and bacterial species were identified. The main outcome measure was the ability to accurately predict infection with ESBL-PE by measures of discrimination and calibration.

**Results:**

Overall, 376 patients (94 patients, 282 controls) were analysed. Performance measures for prediction of ESBL-PE infection of both prediction models indicate adequate measures of calibration, but poor discrimination (area under receiver-operating curve: 0.627 and 0.651). History of ESBL-PE colonisation or infection was the single most predictive independent risk factor for ESBL-PE infection with high specificity (97%), low sensitivity (34%) and balanced positive and negative predictive values (80% and 82%).

**Conclusions:**

Applying published prediction models to institutions these were not derived from, may result in substantial misclassification of patients considered as being at risk, potentially leading to wrong allocation of antibiotic treatment, negatively affecting patient outcomes and overall resistance rates in the long term. Future prediction models need to address differences in local epidemiology by allowing for customisation according to different settings.

## Introduction

Over recent decades, extended-spectrum β-lactamase-producing Enterobacterales (ESBL-PE) rates have increased globally, mainly causing urinary tract and abdominal infections, as well as bacteraemia [1,2]. ESBL-PE have contributed to both healthcare- and community-associated infections and are primarily caused by *Escherichia coli* and *Klebsiella* spp [2-5]. ESBL-PE are able to hydrolyse extended-spectrum penicillins, third generation cephalosporins and monobactams, and also commonly harbour genes conferring resistance to aminoglycosides and fluoroquinolones [1]. With confirmation from the results of a randomised clinical trial, carbapenems are currently regarded as the first-line agents for ESBL-PE bacteraemia [6].

Lack of appropriate ESBL-prediction parameters and time-consuming diagnostic tools for detecting ESBL-PE may result in a steady increase in carbapenem consumption as clinicians may fear administrating antibiotics not providing adequate empirical coverage. While novel diagnostic tools for the detection of ESBL-PE are being developed, e.g. real-time PCR for direct ESBL detection [7], routine laboratory detection is still based on phenotypic tests using chromogenic culture media, followed by a confirmation step, such as the combination disc method [7], together resulting in substantial delay to confirmation. The resulting delay to appropriate therapy might lead to higher morbidity and mortality [8-10], while antibiotic selective pressure from carbapenem overuse fosters Gram-negative bacteria resistant to carbapenems. Thus, simple tools for efficient therapeutic decisions are essential and can complement hospital- and/or ward-specific antibiograms to guide empiric treatment decisions.

Algorithms for the prediction ESBL-PE infection on hospital admission [11] or in patients with bacteraemia [12] have been proposed to inform empiric treatment decisions. Both algorithms include well-established predictors for ESBL-PE related to previous antibiotic exposure, prior hospitalisation, known ESBL-colonisation, age and comorbidities. The authors of the algorithm for prediction of infection with ESBL-PE, which was developed in Italy [11], acknowledged that it reliably identified patients likely to be harbouring ESBL-PE who may need special infection control measures at hospital admission, but that further study was needed to confirm this model's potential as a guide for prescribing empirical antibiotic therapy. Consecutive external validation of this model in the United States (US) showed promising measures of discrimination [13]. However, to be widely applicable, it needs further validation in a region with a lower prevalence of ESBL-PE. We sought to confirm external validity and transferability of these two previously published prediction models, as well as their integral components.

## Methods

### Setting and participants

We performed a retrospective case–control study at the University Hospital Basel, an 813-bed tertiary care academic centre that admits over 30,000 patients per year. Consecutive patients aged ≥ 18 years with detection of ESBL-producing *E. coli* or *K. pneumoniae* isolated from blood cultures between 1 January 2010 and 31 December 2016 were included as cases. For each case, we identified three controls with detection of non-ESBL-producing *E. coli* or *K. pneumoniae* in blood cultures during the same time period, matching for month (± 4 weeks) of detection and Gram-negative bacterial species. We adhered to the Strengthening the Reporting of Observational Studies in Epidemiology (STROBE) guidelines for reporting of observational studies [14].

### Clinical data collection

We retrospectively extracted pertinent clinical data from the patients’ electronic medical records. Patients with documented refusal of general informed research consent were excluded. All study parameters followed strict adherence to the definitions used by Tumbarello et al. [11] and Goodman et al. [12], and with further clarification undertaken with the lead investigators of each study when necessary. The following variables were collected: (i) demographic data, (ii) source of bacteraemia, (iii) presence of chronic indwelling vascular hardware, (iv) history of ESBL-PE colonisation or infection, (v) inpatient and outpatient antibiotic therapy with Gram-negative coverage within the prior 3 and 6 months, (vi) recent hospitalisation, (vii) admission from another healthcare facility (acute care, long-term care or nursing homes), (viii) at least one overnight stay in a hospital in an ESBL high-burden region during the prior 6 months, (ix) underlying diseases and comorbidities on admission based on the Charlson Comorbidity Index of ≥ 4 and (x) urinary catheterisation. Missing data were categorised as ‘negative’ risk factors.

### Definitions

Chronic indwelling vascular hardware was defined as any vascular hardware, except peripheral venous catheters, in place for at least 7 days when the index blood culture was drawn. ESBL history was defined if colonisation or infection with ESBL-PE was detected in any sample within the last 6 months. Antibiotic exposure referred to either an antibiotic therapy with Gram-negative coverage prescribed for at least 6 days within 6 months before the index blood culture (including extended-spectrum penicillins, third/fourth generation cephalosporins, carbapenems, aztreonam, fluoroquinolones or aminoglycosides) or antibiotic therapy with β-lactams or fluoroquinolones for ≥ 48 h within the prior 3 months. Recent hospitalisation was regarded as hospitalisation for > 2 days within 12 months before the current hospital admission. ESBL high-burden regions were defined as countries with a reported percentage of ≥ 20% of either ESBL-producing *E. coli* and/or *K. pneumoniae*. Transurethral or suprapubic catheterisation within 30 days before index blood culture were classified as urinary catheterisation. All bloodstream infections detected from day 3 of hospitalisation or later were considered healthcare-associated, while those diagnosed within the first 48 hours after hospital admission were considered community-associated.

### Microbiological analyses

During the study period the BACT/ALERT 3D and VIRTUO systems (both bioMérieux, Marcy-l’Étoile, France) were used to incubate aerobic and anaerobic blood culture flasks either with charcoal or raisins. Identification of ESBL-PE in bloodstream isolates was performed by standard culture methods in accordance to the guidelines of the Clinical and Laboratory Standards Institute [15]. Species were identified either with MALDI-TOF mass spectrometry (Bruker Daltonics, Bremen, Germany) or biochemically with the VITEK 2 system (bioMérieuex). Specific species within the *K. pneumoniae* group (*K. pneumoniae, K. variicola, K. quasipneumoniae,* and *K. quasivariicola*) could not be separated. The VITEK 2 system was also used for susceptibility testing. ESBL production was based on the detection of resistance to cefpodoxime, ceftriaxone, ceftazidime or aztreonam. Phenotypic confirmation of the ESBL test result was conducted by Etest strips (bioMérieuex) using cefotaxime, ceftazidime or cefepime, each tested with and without clavulanic acid or with Neo-Sensitabs discs (Rosco, Taastrup, Denmark). Minimum inhibitory concentration (MIC) breakpoints were interpreted according to EUCAST guidelines (www.eucast.org). ESBL was reported when at least two of three test substances showed evidence for ESBL.

### Statistical analyses

Baseline characteristics were compared by chi-squared and Fisher’s exact test, where appropriate, for categorical variables and Student’s t-test or Mann–Whitney U test, where appropriate, for continuous variables. To describe the distribution and the strengths of the associations between the components of the two prediction models with ESBL detection among patients with positive blood cultures for *E. coli* or *K. pneumonia,* odds ratios (OR) were calculated applying conditional logistic regression analyses. Stepwise conditional logistic regression using forward and backward selection (with elimination at an α level of 0.05), as well as Akaike information criterion (AIC), were applied to identify variables independently associated with ESBL-PE. To select variables that are most predictive, we further performed least absolute shrinkage and selection operator (lasso) regression, a shrinkage method, shrinking coefficient estimates of predictors with little or no predictive value to zero, an OR of 1 [16], using the Stata module ELASTICREGRESS [17], as well as recursive partitioning algorithms (decision tree statistics) [18] using the Stata module CHAID to conduct chi-squared automated interaction detection [19]. Effect modification by onset of ESBL-PE bacteraemia, community-associated or healthcare-associated, was evaluated using interaction terms and stratified analyses if interaction terms were found to be significant.

Both prediction models were applied in their fully original forms to our dataset. To validate the prediction models, we classified all blood cultures as ‘ESBL-prediction-positive’ or ‘ESBL-prediction-negative’ as suggested [11,12]. Sensitivity, specificity, positive and negative predictive values and the Youden Index were calculated based on this classification. Measures of discrimination and calibration were applied to assess the ability of both prediction models to adequately predict ESBL-PE. We defined discrimination as the ability of the prediction models to separate ESBL-producing *E. coli* or *K. pneumoniae* from non-ESBL-producing *E. coli* or *K. pneumoniae* among patients with positive blood cultures. To quantify discriminative power, the c statistic analogous to the area under the receiver-operating curve (AUC) was calculated. To calculate the Hosmer–Lemeshow statistic and the AUC, we performed logistic regression analyses including the classifying variable, i.e. ‘ESBL-prediction-positive’ or ‘ESBL-prediction-negative’ into the regression models. To internally validate the performance of the prediction model derived from our cohort, k-fold cross-validation for the AUC after fitting the finally selected regression model was performed. Calibration was defined as the measure of how closely predicted values agreed with observed values. Hosmer–Lemeshow goodness-of-fit test was applied to calculate a chi-squared statistic to compare the differences between predicted and actual events, with small values indicating good calibration and values exceeding 20 indicating significant lack of calibration. We generated calibration belt plots for models not assigning dichotomous probabilities (i.e. Score by Tumbarello M et al. [11] and the prediction model derived using stepwise variable selection) [20]. All analyses were performed using Stata version 15.0 (Stata Corp., College Station, Texas, US). p values less than or equal to 0.05 were considered significant.

### Ethical statement

This study was approved by the ethics committee of northern and central Switzerland (Project-ID 2017–01707).

## Results

From 1 January 2010 to 31 December 2016, 98 patients with confirmed ESBL-producing *E. coli* (84%) or *K. pneumoniae* (16%) bacteraemia were identified. One case was excluded because of refusal of informed consent and three were excluded because of a lack of matches, leaving 94 cases who met the eligibility criteria. For each case, three matching controls were identified, resulting in 376 patients included in this analysis.

Evaluating the full cohort, the majority of patients were hospitalised in general medical wards (n = 292, 78%). For 57% (n = 215) of patients, the source of bacteraemia was the urinary tract, followed by an intra-abdominal focus (n = 55, 15%). Of the patients, 10% (n = 38) had an absolute neutrophil count below 1,000 cells/mL. There were 195 (52%) patients hospitalised within the prior 12 months and 40 (11%) had a history of ESBL infection or colonisation. Chronic indwelling vascular hardware was in place in 4% (n = 14) of patients and 20% (n = 75) had urinary catheters. Of the patients, 130 (35%) received antibiotic therapy within the previous 6 months. The mean Charlson Comorbidity Index was 2 (standard deviation: ± 2.25). Cases and controls were balanced regarding baseline characteristics (Table 1).

**Table 1:** Demographic and clinical characteristics of patients by extended-spectrum β-lactamase-producing Enterobacterales infection status, University Hospital Basel, Switzerland, January 2010–December 2016 (n = 376)

Characteristic	Cases (ESBL-positive) (n = 94)		Controls (ESBL-negative) (n = 282)		p value^a^
	n	%	n	%	
Age (years) (median, IQR)	69	57–76	73	59–81	0.060
Female	48	51	161	57	0.338
Male	46	49	121	43	
Ward					0.642
- Medicine	74	79	218	77	
- Surgery	9	10	38	13	
- Gynaecology	2	2	7	2	
- Intensive care unit	9	10	19	7	
Source of bacteraemia					0.253
- Urinary tract	47	50	168	60	
- Intra-abdominal	12	13	43	15	
- Immunosuppressive diseases	12	13	25	9	
- Pulmonary	3	3	6	2	
- Gynaecologic diseases	2	2	2	1	
- Indwelling hardware	4	4	10	4	
- Wound infection	3	3	6	2	
- Other	0	0	6	2	
- Unknown	11	12	16	6	

ESBL: extended-spectrum β-lactamase; IQR: interquartile range.

**^a^** p values were calculated by chi-squared or Fisher’s exact test (as appropriate) for categorical variables and by the Student’s t-test or Mann–Whitney U test (as appropriate) for continuous variables. For categorical variables with more than two categories, we present overall p values.

Many variables included in the prediction models were associated with ESBL-PE infection in our cohort according to univariable regression analysis, i.e. recent hospitalisation, admission from another healthcare facility, previous therapy with β-lactams and/or fluoroquinolones, urinary catheterisation, history of ESBL-PE-colonisation/infection or any antibiotics in the prior 6 months (Table 2). All multivariable conditional logistic regression models using stepwise forward and backward selection, as well as the AIC for variable selection, selected history of ESBL-PE colonisation or infection, admission from another healthcare facility, and antibiotic therapy with β-lactams or fluoroquinolones lasting > 48 h during the 3 months preceding admission as independent predictors for ESBL-PE bacteraemia in our cohort (Table 2). Variable selection using lasso regression and recursive partitioning algorithms revealed history of ESBL-PE colonisation or infection as the only variable predictive of ESBL-PE bacteraemia. We identified no significant effect modification, except for antibiotic therapy with β-lactams or fluoroquinolones lasting > 48 h during the 3 months preceding admission by onset of ESBL-PE bacteraemia (Table 3). Stratified analyses revealed this exposure being associated with ESBL-PE bacteraemia in community-acquired (OR: 8.32, 95% CI: 3.62–19.11, p < 0.001) but not healthcare-acquired infection (OR: 3.32, 95% CI: 0.65–16.99, p = 0.150).

**Table 2:** Univariable and multivariable analyses of established predictors of extended-spectrum β-lactamase-producing Enterobacterales infection, University Hospital Basel, Switzerland, January 2010–December 2016 (n = 376)

Parameter	Cases (ESBL-positive) (n = 94)		Controls (ESBL-negative) (n = 282)		Univariable analyses			Multivariable analyses^a^		
	n	%	n	%	OR	95% CI	p value	OR	95% CI	p value
**Parameters by Tumbarello et al. [** 11 **]**										
Recent hospitalisation^b^	60	64	135	48	2.16	1.27–3.69	**0.005**	NS	NA	NA
Admission from other healthcare facility	17	18	26	9	2.18	1.12–4.25	**0.023**	3.07	1.37–6.88	**0.007**
Antibiotic therapy with β-lactams or fluoroquinolones^c^	60	64	74	26	4.96	3.02–8.16	**< 0.001**	4.10	2.17–7.74	**< 0.001**
Urinary catheterisation^d^	28	30	47	17	2.17	1.25–3.77	**0.006**	NS	NA	NA
Charlson Comorbidity Index ≥ 4	16	17	44	16	1.10	0.59–2.05	0.767	NS	NA	NA
Age ≥ 70 years	47	50	165	59	1.21	0.75–1.95	0.444	NS	NA	NA
**Parameters by Goodman et al. [** 12 **]**										
History of ESBL-PE colonisation/infection^e^	32	34	8	3	18.06	7.02–46.47	**< 0.001**	15.32	5.52–42.53	**< 0.001^f^**
Hospitalisation in ESBL high-burden region^e,g^	1	1	2	1	1.50	0.14–16.54	0.741	NS	NA	NA
Chronic indwelling vascular hardware^h^	21	22	45	16	1.61	0.86–3.00	0.136	NS	NA	NA
Any antibiotic exposure within last 6 months^i^	56	60	74	26	5.85	3.21–10.64	**< 0.001**	NS	NA	NA
Age ≥ 43 years	87	93	260	92	1.25	0.49–3.20	0.647	NS	NA	NA

CI: confidence interval; ESBL: extended-spectrum β-lactamase; ESBL-PE: extended-spectrum β-lactamase-producing Enterobacterales; OR: odds ratio NA: not applicable; NS: not selected.

^a^ Multivariable analyses included all variables presented in the table, only variables chosen by stepwise logistic regression using stepwise forward and backward selection, as well as Akaike information criterion (AIC), are presented in the table.

^b^ More than 2 days within 12 months before index hospitalisation.

^c^ Lasting > 48 h during the 3 months preceding admission.

^d^ Within 30 days before index blood culture.

^e^ Within 6 months before index hospitalisation.

^f^ Selected by lasso regression and recursive partitioning algorithms (decision tree statistics).

^g^ India (1), France (1) and North Macedonia (1).

^h^ Central venous catheters (including peripherally inserted central catheter (PICC)-lines, ports, pace makers) and central dialysis catheters.

^i^ Extended-spectrum penicillins, third/fourth generation cephalosporins, carbapenems, aztreonam, fluoroquinolones and aminoglycosides.

Bold print indicates significant p values.

**Table 3:** Effect modification onset of infection (community-associated vs hospital-associated) for established predictors of extended-spectrum β-lactamase-producing Enterobacterales infection, University Hospital Basel, Switzerland, January 2010–December 2016 (n = 376)

Parameter	Effect modification by onset of infection (community-associated vs hospital-associated)		
	OR^a^	95% CI	p value
Recent hospitalisation^b^	2.35	0.81–6.79	0.116
Admission from other healthcare facility	NA	NA	NA
Antibiotic therapy with β-lactams or fluoroquinolones^c^	3.84	1.17–12.56	**0.026**
Urinary catheterisation^d^	2.26	0.68–7.50	0.181
Charlson Comorbidity Index ≥ 4	1.53	0.32–7.39	0.598
Age ≥ 70 years	0.71	0.25–1.97	0.507
History of ESBL-PE colonisation/infection^e^	1.06	0.20–5.62	0.943
Hospitalisation in ESBL high-burden region^e,f^	NA	NA	NA
Chronic indwelling vascular hardware^g^	1.98	0.58–6.78	0.278
Any antibiotic exposure within last 6 months^h^	2.50	0.80–7.81	0.114
Age ≥ 43 years	1.12	0.15–8.31	0.910

CI: confidence interval; ESBL-PE: extended-spectrum β lactamase-producing Enterobacterales; NA: not applicable; OR: odds ratio.

^a^ Odds ratios and respective 95% CIs represent interactions terms.

^b^ More than 2 days within 12 months before index hospitalisation.

^c^ Lasting > 48 h during the 3 months preceding admission.

^d^ Within 30 days before index blood culture.

^e^ Within 6 months before index hospitalisation.

^f^ India (1), France (1) and North Macedonia (1).

^g^ Central venous catheters (including peripherally inserted central catheter (PICC)-lines, ports, pace-makers) and central dialysis catheters

^h^ Extended-spectrum penicillins, third/fourth generation cephalosporins, carbapenems, aztreonam, fluoroquinolones and aminoglycosides.

Bold print indicates significant p values.

Performance measures for prediction of ESBL-PE infection of both algorithms in our cohort as published in the original papers are shown (Table 4), revealing adequate measures of calibration, but poor discrimination (AUC 0.627 and 0.651). In addition, performance characteristics of all variables selected by stepwise selection and selection using the lowest AIC value (history of ESBL-PE colonisation or infection, admission from another healthcare facility, and antibiotic therapy with β-lactams or fluoroquinolones lasting > 48 h during the 3 months preceding admission), as well as history of ESBL-PE colonisation or infection, selected by lasso regression and recursive partitioning algorithms was performed (Table 4). The model based on variables selected by stepwise selection and the lowest AIC value showed the most favourable performance characteristics in terms of AUC and Youden’s Index, even after k-fold cross-validation. The Figure shows calibration belt plots for the score by Tumbarello et al. [11] (A) and the prediction model derived using stepwise variable selection) (B).

**Table 4:** Performance measures for prediction of extended-spectrum β-lactamase-producing Enterobacterales infection, University Hospital Basel, Switzerland, January 2010–December 2016 (n = 376)

Performance measure	Prediction of infection/colonisation with ESBL-PE on hospital admission	Prediction ESBL-PE in patients with bacteraemia		
	Score by Tumbarello et al. [11]	Decision tree by Goodman et al. [12]^a^	Prediction model derived using stepwise variable selection^b^	Known history of ESBL-PE colonisation/infection^c^
Sensitivity	67.0%	33.0%	74.5%	34.0%
Specificity	52.8%	97.2%	67.7%	97.2%
Positive predictive value^d^	32.1%	79.5%	43.5%	80.0%
Negative predictive value^d^	82.8%	81.3%	88.8%	81.5%
Youden-Index	0.2	0.3	0.4	0.3
Hosmer–Lemeshow statistic	1.96 (p = 0.855)	4.43 (p = 0.816)	1.61 (p = 0.657)	NA
Area under the curve (AUC)	0.627^e^	0.651^f^	0.759 (0.710^g^)	0.656 (0.598^g^)

ESBL-PE: extended-spectrum β lactamase-producing Enterobacterales; NA: Not applicable.

^a^ For calculations of the Hosmer–Lemeshow statistic and the area under the curve (AUC), logistic regression analyses including the classifying variable, i.e. ESBL-prediction positive or negative, into the regression model was performed.

^b^ Including history of ESBL-PE colonisation or infection, admission from another healthcare facility, and antibiotic therapy with β-lactams or fluoroquinolones lasting > 48 h during the 3 months preceding admission.

^c^ Selected by lasso regression and recursive partitioning algorithms (decision tree statistics).

^d^ Both the positive and the negative predictive values are influenced by the study design and thus have to be interpreted with caution.

^e^ Ranging from 0.83 (derivation cohort) to 0.92 (validation cohort).

^f^ Reported as 0.77 in the original publication (for the final decision tree and following cross-validation).

^g^ k-fold cross-validation.

**Figure:**
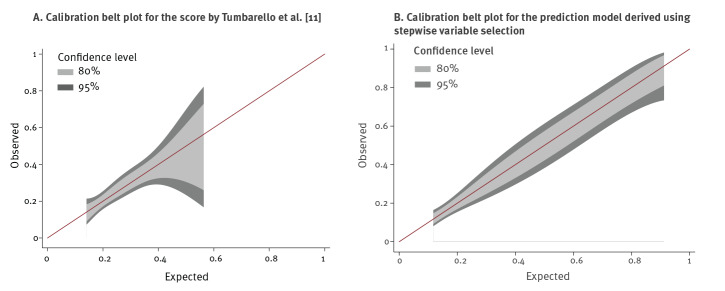
Calibration belt plots for the score by Tumbarello et al. [11] (A) and the prediction model derived using stepwise variable selection (B) applied to the cohort investigated in this extended-spectrum β-lactamase-producing Enterobacterales (ESBL-PE) study, University Hospital Basel, Switzerland, January 2010–December 2016 (n = 376)

## Discussion

Independent and external validation of two previously published prediction models for detection of infection with ESBL-PE revealed poor predictive accuracy for ESBL-PE infection in a low-endemic setting, questioning their transferability to other settings. Among all integral components of both prediction scores, we identified a number of risk factors associated with ESBL-PE infection, including recent hospitalisation, urinary catheterisation, previous antibiotic therapy, admission from another healthcare facility and history of ESBL-PE colonisation/infection.

These variables have also been identified in previous studies investigating risk factors for ESBL-PE infection [21-24]. Other variables such as the Charlson Comorbidity Index, chronic vascular hardware, hospitalisation in a high-burden region or age were not associated with an increased risk of ESBL-PE infection, possibly pointing to the changing epidemiology of ESBL-PE and their increasing prevalence in the community [25], thus resulting in infections in younger and previously healthy patients. This hypothesis is supported by exposure to β-lactams or fluoroquinolones > 48 h during the 3 preceding months being related with community-associated infection rather than hospital-associated infection in the analyses of effect modification in our cohort. This in turn suggests that selective pressure may be the main driver in a colonised population. The number of patients previously hospitalised in an ESBL-high-burden region was low in our cohort (three patients: 1 case and 2 controls) making it difficult to draw inferences regarding the external validity of this risk factor. Multivariable analysis using different stepwise variable selection approaches resulted in a prediction model showing increased but still moderate discriminative power in our cohort consisting of three of the assessed parameters (admission from another healthcare facility, antibiotic therapy with β-lactams or fluoroquinolones > 48 h during the 3 preceding months, and history of ESBL-PE colonisation or infection), which were independently predictive of ESBL-PE bacteraemia. However, we acknowledge that the higher discriminatory power detected in this model may be explained by the calculation of the AUC for a multivariable regression model rather than for a derived score, possibly resulting in a more favourable AUC and thus hampering comparisons of the AUCs between the model derived from our cohort and the previously published prediction scores. Internal validation of this prediction model applying k-fold cross-validation revealed a lower AUC, as did history of ESBL-PE colonisation or infection when compared with the originally fitted models, thus uncovering the issue of overfitting as a potential shortcoming of prediction models derived from one cohort. However, we cannot rule out that the lower AUC may also be related to the relatively small sample size in our cohort. In line with our results, a Dutch study by Platteel et al. also identified both previous ESBL-carriage and previous hospital admission as independent risk factors for ESBL-carriage. The model, however, exhibited poor discrimination with an AUC of 0.64 [26]. The authors therefore concluded that a clinically useful prediction rule for ESBL carriage could not be developed.

History of ESBL-PE infection or colonisation revealed the highest association with ESBL-PE bacteraemia in our cohort and was the only variable selected by lasso regression and recursive partitioning algorithms, both aiming to enhance the prediction accuracy and interpretability of a statistical model. While the discriminatory power of history of ESBL-PE infection or colonisation was low (however, similar to the discriminatory power of the two externally validated prediction models), its positive and negative predictive value, as well as its specificity revealed favourable results. History of ESBL-PE colonisation or infection is a well-known risk factor for developing serious infections [27,28], which is easy to ascertain by medical chart review. Our findings suggest that it may present an easily identifiable proxy to predict ESBL-PE infection, its high specificity (corresponding to a low false-positive rate) allowing for the identification of patients requiring carbapenems in the case of suspected bacteraemia with high confidence.

Differences in the epidemiology of ESBL-PE between all three countries may in part explain the poor performance of both prediction models, with both Italy and the US reporting a higher ESBL prevalence than Switzerland. In Switzerland, ca 10% of all clinical *E. coli* strains and *K. pneumoniae* strains are reported as being resistant to third generation cephalosporins, suggesting the presence of ESBLs [29]. In Italy, where the score by Tumbarello et al. [11] was developed, the proportions of *E. coli* and *K. pneumoniae* resistant to third generation cephalosporins were 61.6% and 38.9%, respectively in 2008, which was when the study was performed [30]. In the US, where the score by Goodman et al. [12] was developed, the proportions of ESBL-producers have been reported as 14% for *E. coli* and 23% for *K. pneumoniae* causing healthcare-associated infections [31]. Future prediction models need to address differences in local epidemiology, possibly by allowing for adaption of specific variables according to the geographic setting. Adapting variables to specific settings may, however, prove to be challenging, as a recent study [32] aiming to predict the probability of colonisation with carbapenem-resistant organisms by including 125 variables and machine learning methods at a single institution, representing a constricted geographic setting, failed to derive a clinically useful prediction model. This points to intrinsic difficulties in generating such models, even when considering a large amount of variables deriving from one single setting and thus not subjected to differences in local epidemiology [32]. Applying published prediction models to institutions where these were not derived from may result in substantial misclassification of patients considered as being at risk, potentially leading to the incorrect allocation of antibiotic treatment and infection control measures, ultimately negatively affecting both patient outcomes and overall resistance rates in the long term. Such considerations are of importance on a European level and beyond. Based on the 2017 EARS-Net-report [33], in the European Union (EU) and European Economic Area (EEA), the population-weighted mean resistance percentage for third-generation cephalosporin resistance (mainly reflecting ESBL-production) of *E. coli* and *K. pneumoniae* was 14.9% and 31.2%, respectively. However, the range of the resistance percentage is considerable for both *E. coli* (5.9% in Norway to 41.3% in Bulgaria) and *K. pneumoniae* (4.6% in Finland to 76.3% in Bulgaria). In line with differences in resistance proportions, it is reasonable to assume that risk factors for ESBL-PE infection may differ substantially between different countries and thus heavily influence performance of prediction tools developed in other settings. Such differences are unlikely to be merely overcome by deriving prediction algorithms from larger datasets from multiple institutions and countries, but call for strategies including cross-validation on both a national and institutional level, as well as the inclusion of variables allowing for specific local adaption. Such a strategy was chosen for the development of the simplified acute physiology score 3 (SAPS III) [34]. This model for predicting hospital mortality in intensive care unit (ICU) patients includes both well-established parameters for the prediction of ICU mortality but also allows for customisation according to different regions thereby resulting in improved calibration [35]. Novel statistical approaches, including machine learning algorithms may enable the identification of more useful prediction tools in the future, especially when applied to large electronic datasets.

Our study has a number of limitations. First, this is a retrospective study collecting data from medical records. Thus, certain data were not systematically evaluated at hospital admission, i.e. recent hospitalisation in a high-burden-region or recent antibiotic therapy, as would be the case in a prospective study using standardised interview protocols to determine the presence or absence of such risk factors. Missing data were categorised as ‘negative’ risk factors and might have led to an underestimation of association of these parameters with ESBL-PE infection, possibly explaining the poor performance of both prediction models. However, it is standard practice to collect information on all the clinical variables considered in this study, including recent hospitalisation in a high-burden-region or recent antibiotic therapy. Second, the study design also influenced the outcome of positive and negative predictive values, such that what might be an accurate predictive tool in our institution may perform sub optimally in areas with higher or lower prevalence of ESBL-PE. Both the positive and the negative predictive values therefore need to be interpreted with caution. Third, performing this study in a single academic tertiary care centre indicates inclusion of a high number of patients with complex underlying medical conditions and therefore limits the applicability of the findings to other healthcare facilities. Further studies are needed to review adaptability of the algorithms to non-tertiary care facilities since recent data reported rising rates of community-associated ESBL-PE infections in small community hospitals [36]. Fourth, because of low incidence of ESBL-PE in our institution, we restricted data collection to patients with infections caused by either ESBL-producing *E. coli* or *K. pneumoniae*; thus, our findings are not generalisable to other ESBL-producing Enterobacterales. Fifth, only patients with ESBL-PE in blood cultures were included in our study, while the prediction score by Tumbarello et al. was derived from the detection of ESBL-PE from any site. Sixth, we acknowledge that there is a lack of recommendations on how to formally validate decision trees; thus, our approach is not based on standardised criteria.

In summary, poor accuracy of the two prediction algorithms in our study question their transferability to other settings. Applying published prediction models to institutions where these were not derived from, may result in substantial misclassification of patients considered as being at risk, potentially leading to the incorrect allocation of antibiotic treatment, ultimately, negatively affecting both patient outcomes and overall resistance rates in the long term. Future prediction models need to address differences in local epidemiology by allowing for customisation according to different settings. In the meantime, in terms of specificity and positive and negative predictive values, a history of ESBL-PE infection or colonisation seems to be the single most reliable predictor of ESBL-PE bacteraemia. Admission from another healthcare facility and antibiotic therapy with β-lactams or fluoroquinolones lasting > 48 h during the prior 3 months may also enhance the discriminatory power of prediction algorithms and future risk stratification models.
